# Lateralized Cerebellar Contributions to Word Generation: A Phonemic and Semantic Fluency Study

**DOI:** 10.3233/BEN-2010-0269

**Published:** 2010-08-16

**Authors:** Tom A. Schweizer, Michael P. Alexander, B. A. Susan Gillingham, Michael Cusimano, Donald T. Stuss

**Affiliations:** ^1^Division of NeurosurgerySt. Michael's HospitalTorontoCanada; ^2^Keenan Research Centre of the Li Ka Shing Knowledge Institute at St. Michael’s HospitalTorontoCanada; ^3^Faculty of MedicineDepartment of SurgeryDivision of NeurosurgeryUniversity of TorontoTorontoCanada; ^4^Rotman Research Institute at BaycrestTorontoCanada; ^5^Harvard Medical School and Beth Israel Deaconess Medical Centre (Neurology)BostonUSA; ^6^Faculty of Medicine (Neurology Rehabilitation Sciences) and PsychologyUniversity of TorontoTorontoCanada

## Abstract

Impairment on verbal fluency tasks has been one of the more consistently reported neuropsychological findings after cerebellar lesions, but it has not been uniformly observed and the possible underlying cognitive basis has not been investigated. We tested twenty-two patients with chronic, unilateral cerebellar lesions (12 Left, 10 Right) and thirty controls on phonemic and semantic fluency tasks. We measured total words produced, words produced in the initial 15 seconds, errors and strategy switches. In the phonemic fluency task, the right cerebellar lesion (RC) group produced significantly fewer words compared to the left cerebellar lesion (LC) group and healthy controls, particularly over the first 15 seconds of the task with no increase in errors and significantly fewer switches over the entire task. In the semantic fluency task there was only a modest decrease in total words in the RC group compared to controls. RC lesions impair fluency with many of the same performance characteristics as left prefrontal lesions. This supports the hypotheses of a prefrontal-lateral cerebellar system for modulation of attention/executive or strategy demanding tasks.

## 1. Introduction

Tests of word generation are commonly used in neuropsychological assessment. There are two types of tasks. In one type, subjects generate a response word, for example a verb (action) related to a provided target word, for example a noun (object). In the other type, subjects produce exemplars from a specified category with only the structure of the category to guide them. To distinguish these, the former is referred to as "generation" tasks and the latter as "(verbal) fluency" tasks. Semantic fluency is the production of words within specified semantic categories (animals, tools, foods, etc.); phonemic fluency is the production of words that begin with specified letters, usually with restrictions – no proper names, no sequential derivations. Fluency tasks are useful because, assuming preservation of confrontation naming, they assess both associative processes – phonological and semantic – and strategic abilities [1,16,20,27,29]. When one associative *cluster* (farm animals or words beginning with 'fa-"), is exhausted, the solution requires *shifting* to another (African animals or words beginning with 'fi-'). As the list unfolds, mentally tracking and monitoring (working memory) responses is required to avoid

ISSN 0953-4180/10/$27.50 © 2010 – IOS Press and the authors. All rights reserved

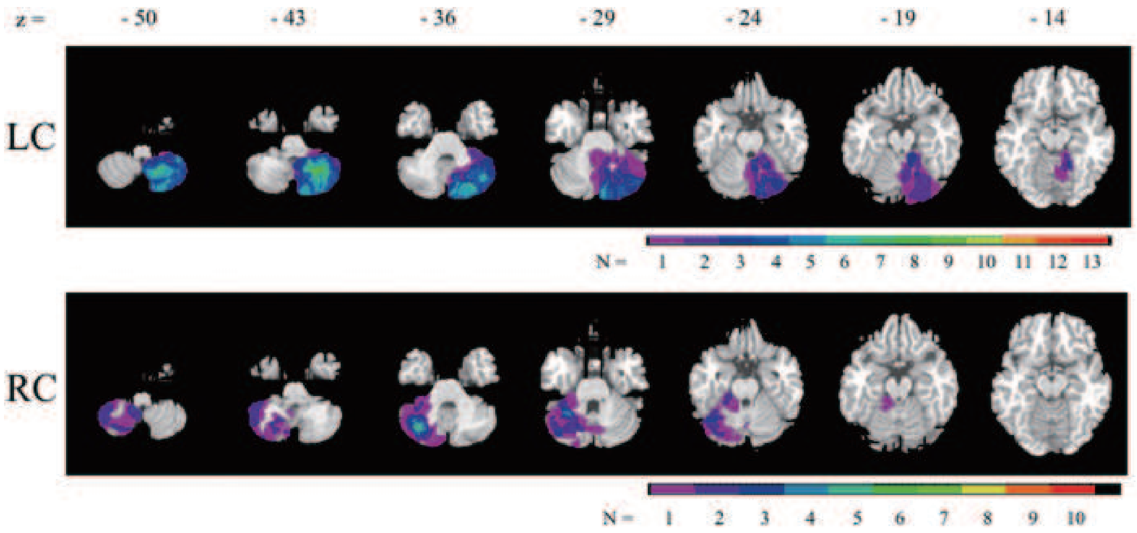

Fig. 1. Lesion overlaps of the individual patients in each of the patient groups using radiological conventions on a standard image template provided by MRIcro software. The z coordinates for each axial slice represent the distance from the anterior commisure ($z = 0$) in millimeters. The corresponding colour bars represent areas of greater overlap (i.e., more patients) moving from left to right.

repetitions and errors. Across the task, attention must be sustaining to the overall goal and constraints.

Much of the value to Neuropsychology comes from these multi-dimensional requirements. There is a very large literature on differential effects on specific dimensions of lesions in different areas of cortex. For instance, lesions in a broad region of the left hemisphere and the right frontal lobe may impair verbal fluency, some due to direct damage to language systems or the auditory-verbal short term memory component of working memory, but others presumably due to damage to strategic or monitoring requirements. Prefrontal regions play a critical role (see Henry and Crawford, 2004 for meta-analysis). Left prefrontal lesions are particularly likely to impair fluency: reduction in total words generated [27] and deficient switching as opposed to smaller clusters with left temporal lesions [29]. Prefrontal regions manage switching in verbal fluency by disengaging from an active search schema [28]. Monitoring may recruit right frontal regions [27].

Evidence for an effect of cerebellar lesions on fluency (and perhaps generation) emerged most convincingly in a seminal paper by Schmahmann and Sherman [22]. Since then, there have been other investigations of both generation and fluency in patients with a variety of cerebellar disorders [13,15,18]. Deficits in fluency have been consistently reported, usually but not uniformly following right cerebellar injuries. There is also convergent support for the role of both the left prefrontal cortex and the right cerebellum for both forms of word generation tasks from neuroimaging activation studies in normal healthy individuals [5,10]. The initial observation that the cerebellum is activated by generation tasks actually came from the incidental observation of right cerebellum activation (in parallel with left frontal activation) in a verb generation PET study [17]. The neuroimaging studies of cerebellar activation in cognitive tasks were exhaustively reviewed by Stoodley and Schmahmann (2008). The meta-analysis of language studies was dominated by studies of fluency and generation: "word/letter generation, word stem completion, ... verbal fluency". The critical region identified in the language studies was right hemisphere – lobule VI and crusI/crusII – seen in their Fig. 1b and 1c.

So, while there is evidence from multiple sources to support a role for the right cerebellum in fluency, there is only one report [13] that has explored the role of cerebellar localization on dimensions of performance. If there are discrete effects on clustering, sustaining attention, and monitoring, they have not been completely described. The available studies are heterogeneous in structure – some acute, some late, some with only focal lesions, some mixed with degenerative disorders – making general conclusions tentative. To clarify some of the remaining questions about the cerebellum and fluency, we investigated verbal fluency as part of a larger study of cognition in a group of patients with focal cerebellar injuries, carefully screened for any complicating co-morbidity and only tested in a late, stable epoch after injury.

## 2. Methods

### 2.1. Participants

Twenty-two patients (age range 24–78, mean 54.41; education range 7–21, mean 13.82) with focal cerebellar lesions (11 stroke, 9 post-operative benign tumour resections, 2 hemorrhage) were recruited from three separate neurology and neurosurgery centres (Baycrest Centre and St. Michael's hospital in Toronto and Beth Israel Deaconess Medical Center in Boston) and tested at least 90 days post-injury (range 90–1035; mean 222). For purposes of a language laterality study it was important to include only right-handed patients. Thirty control participants (CTL; age range 22–80, mean 52.27; education range 12–24, mean 15.90) were recruited either from the research participant pool at Baycrest Centre or through advertisement at Beth Israel Deaconess Medical Centre. We excluded patients who were dysarthric and none of the patients or controls had a previous stroke, traumatic brain injury, any other neurological disease, or any axis-I DSM diagnosis.

Lesions were identified on either CT or 1.5T MRI scans. All lesions were unambiguously unilateral (12 Left [LC], 10 Right [RC]). No patient had imaging or clinical evidence for a lesion above the mid-pons. Brainstem lesions at or above this level potentially involve important cortical-cerebellar networks. Lesions were drawn on standard brain templates using MRIcro software [19]. Lesion volumes were calculated as a percentage of total volume of the template using inhouse software (RRI) comparing the number of 1 mm $\times$ 1 mm pixels of a region of interest lesion mask drawn in MRIcro to the number of 1 mm $\times$ 1 mm pixels of the brain template on which the lesion was drawn. See Fig. 1 for lesion overlaps of patients grouped by hemisphere of damage. Lesion locations were extracted by mapping on to the regional cerebellar template recently published by Schmahmann and colleagues [21].

The research ethics board at each of the three individual sites approved this study and written consent was obtained from all participants prior to inclusion according to the Declaration of Helsinki.

### 2.2. Verbal fluency tasks

Phonemic fluency (F, A, S): say as many words as possible that began with each of the three letters for one minute per letter, with the exclusion of using suffixes or proper nouns as word-generating strategies, total summed across all three letters.

Semantic fluency (animals): produce as many animal names as possible in one minute, allowing any type (farm, game, birds, fish, etc).

### 2.3. Dependent Measures

For each of the fluency tasks, dependent measurements were chosen to reflect language functioning, the ability to initiate and sustain a task, and strategy application.

<u>Fluency</u>: a) total correct, b) total in the first 15 seconds and c) total in the last 45 seconds.

<u>Errors</u>: a) perseverations (repetition of a word within the same trial); b) proper noun (names of people, places, or brands, excluding those brands that have entered the common lexicon such as Ski-doo); c) suffix (repetition of a word with a different ending, such as bake and baking); d) non-word; e) intrusions (words that do not belong to the target category. The final dependent measure was the sum total of errors from all categories across all three of the letter trials and over the single semantic trial.

<u>Switches</u>: A measure of strategic word generation was adapted from Laine and Niemi [12] and Troyer et al. [29]. Within each letter trial, consecutively generated words were grouped based upon the following criteria: i) began with at least the first two same letters (e.g., <u>sm</u>all, <u>sm</u>art); ii) appeared in alphabetical order (e.g., <u>aa</u>rdvark, <u>ab</u>le, <u>ac</u>e); iii) were synonymous (e.g., slender, slim); iv) rhymed, or were different only by the exchange of one letter (e.g., b<u>a</u>nd, b<u>o</u>nd); v) belonged to the same semantic category (e.g., face, finger). The same criteria were used for the semantic category trial with the exception of (i) and (ii) being based upon just the first letter of the word, and criteria (v) based upon sub-categories of animals divided by type (e.g., farm animals, sea animals), or geographic location (e.g., found on particular continents). The transition between these word groupings, including intervening single words, was labeled as a switch. These switches were added over all three of the letter trials, and over the single semantic trial, for a measure of total switches.

### 2.4. Statistics

Patient groups and the CTL group were compared using ANOVA with an $\alpha$ level of .05, and Tukey's post hoc for follow-up analysis.

## 3. Results

See Table 2 for the demographic data for all participants. There were no significant differences between

Table 1
Patient injury information. Lesion volume represents the percentage of 1 mm voxels of lesion compared to the total number of 1 mm voxels of the entire cerebellum using a standard MRIcro template illustrated in Fig. 1

| Subject | Etiology | Time since injury (months) | Lesion volume (%) |
|---|---|---|---|
| Left Cerebellum | | | |
| 1 | Tumour | 3 | 8.71 |
| 2 | Stroke | 9 | 7.47 |
| 3 | Stroke | 3 | 1.51 |
| 4 | Stroke | 3 | 15.26 |
| 5 | Stroke | 3 | 21.31 |
| 6 | Stroke | 4 | 20.12 |
| 7 | Stroke | 4 | 2.60 |
| 8 | Stroke | 11 | 13.99 |
| 9 | Tumour | 16 | 5.72 |
| 10 | Tumour | 26 | 5.19 |
| 11 | Tumour | 3 | 2.01 |
| 12 | Tumour | 3 | 9.21 |
| Right Cerebellum | | | |
| 13 | Stroke | 3 | 4.10 |
| 14 | Stroke | 4 | 1.34 |
| 15 | Stroke | 3 | 3.40 |
| 16 | Stroke | 4 | 1.23 |
| 17 | Tumour | 6 | 5.41 |
| 18 | Tumour | 13 | 2.18 |
| 19 | Haemorrhage | 3 | 4.55 |
| 20 | Tumour | 33 | 5.36 |
| 21 | Haemorrhage | 35 | 1.01 |
| 22 | Tumour | 23 | 7.65 |

Table 2

| | N (Male) | Hand | Age | Education | NART | BNT |
|---|---|---|---|---|---|---|
| LC | 12 (3) | 12R | 52.83 | 13.00 | 106.31 | 57.09 |
| | | | (16.65) | (2.86) | (9.38) | (1.51) |
| RC | 10 (7) | 10R | 56.30 | 14.80 | 105.18 | 55.11 |
| | | | (12.82) | (3.99) | (10.59) | (6.03) |
| CTL | 30 (19) | 30R | 52.27 | 15.90 | 109.10 | 56.83 |
| | | | (15.14) | (3.01) | (7.91) | (2.93) |

groups on age or estimated IQ (NART-R). Confrontation naming (Boston Naming Test, BNT) was equivalent in patient groups and controls, and there was no correlation between BNT and either phonemic or semantic fluency total scores. The CTL group was significantly more educated than the LC patients [$F_{(2,51)} =$ 3.588, $p <$ 0.04].

There was no difference in mean cerebellar lesion size (volume) between the right and left lesion groups [$F_{(2,55)} =$ 2,580, $p <$ 0.2]. There was no difference in any measure between the chronic (mostly tumour cases) and the relatively acute (mostly stroke cases) groups but the numbers were small precluding comprehensive statistical analyses. The statistical results did not change with the elimination of cases with trivial medullary encroachment after PICA strokes.

### *3.1. Phonemic fluency*

Total: RC patients generated fewer total words over the three letters than both the LC and CTL groups [$F_{(2,51)} =$ 3.3.369, $p <$ 0.05] (see Fig. 2a); initial 15 seconds: the RC group produced significantly fewer words in the first 15 seconds [$F_{(2,51)} =$ 3.306, $p <$ 0.05] than the CTL group only; final 45 seconds: there was a trend for the RC group to produce significantly fewer words than the CTL group only [$F_{(2,51)} =$ 2.735, $p <$ 0.08] (see Fig. 2b).

Four patients with the large lesions in right Crus II were quite impaired on each measure, but the number of patients with lesions in discrete regions of the cerebellum was too small for statistical analysis.

Errors: There were very few errors, essentially all perseverations, and there were no significant differ-

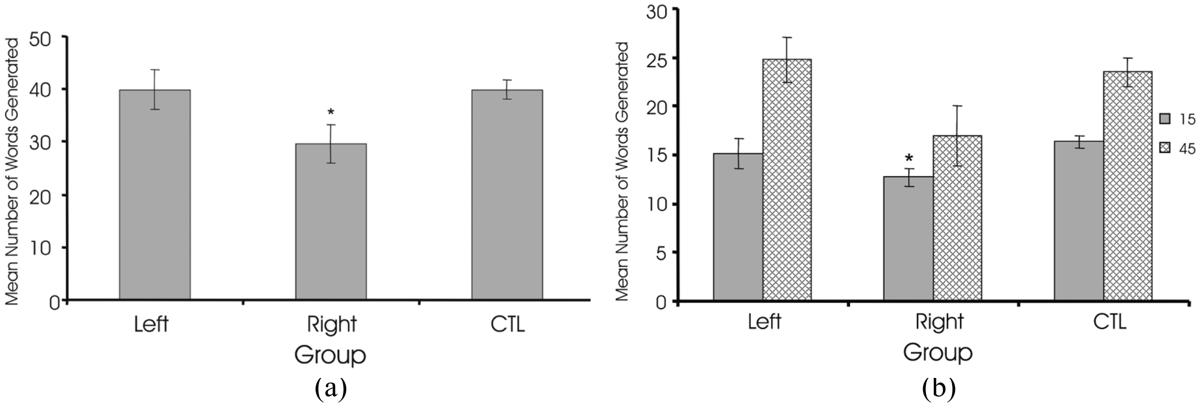

Fig. 2. (a) Word generation summed over three trials in the letter-based fluency task. (b) Word generation summed over three trials in the letter-based fluency task, separated into time intervals defined by the first 15 seconds and the last 45 seconds.

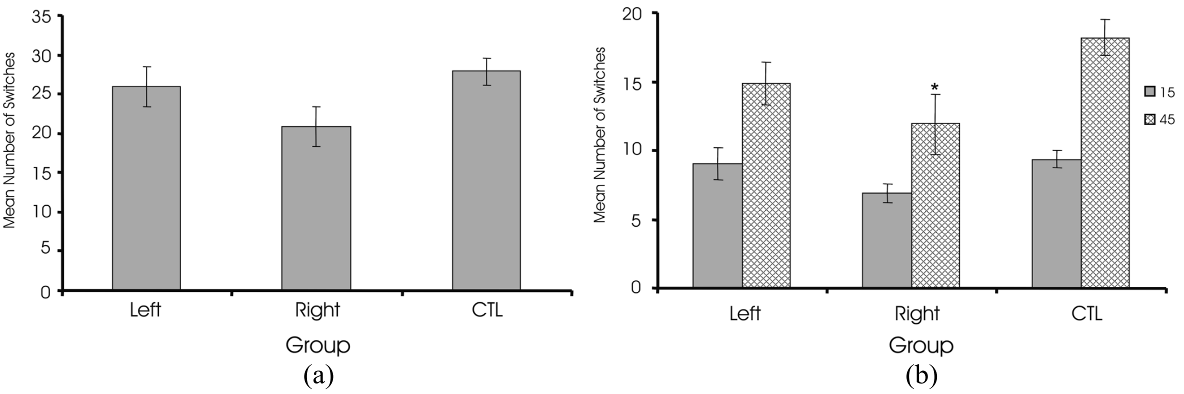

Fig. 3. (a) The number of switches summed over three trials in the letter-based fluency task. (b) The number of switches summed over three trials in the letter-based fluency task, separated into time intervals defined by the first 15 seconds and the last 45 seconds.

ences between groups in errors [$F_{(2,51)} = 2.237$, $p < 0.2$].

Switches: There were no between group differences in the number of switches over the full minute [$F_{(2,51)} = 2.392$, $p < 0.2$] (see Fig. 3a). The RC group had fewer switches than just the CTL group in the last 45 seconds [$F_{(2,51)} = 3.578$, $p < 0.04$] (see Fig. 3b). The LC group did not differ significantly from CTLs on either measure.

### *3.2. Semantic fluency*

Total: the RC group generated fewer words over the full minute than the CTL group [$F_{(2,51)} = 3.167$, $p < 0.05$] (see Fig. 4a) without any significant differences in the first 15 seconds or the last 45 (see Fig. 4b).

Errors: There was essentially none.

Switches: There was no difference between groups over the whole minute trial [$F_{(2,51)} = 1.283$, $p < 0.3$].

## 4. Discussion

The RC group had impaired verbal fluency in both tasks when compared to the LC group and controls for phonemic and to controls only for semantic. In the phonemic fluency task analysis of performance reveals some underlying executive and attention deficits. The RC group was specifically impaired over the first 15 seconds, a phenomenon that we have characterized as either task setting in patients with left ventrolateral prefrontal lesions or deficient initial energization to task in patients with dorsomedial prefrontal lesions [2]. If perseverative errors are a measure of performance monitoring then neither the RC nor LC group had monitoring deficits. The RC group utilized fewer switches over the entirety of the task similar to what was observed after left prefrontal lesions. The precise localization of relevant RC lesion(s) is not certain, but the similarity in impairment profiles supports a functional interaction

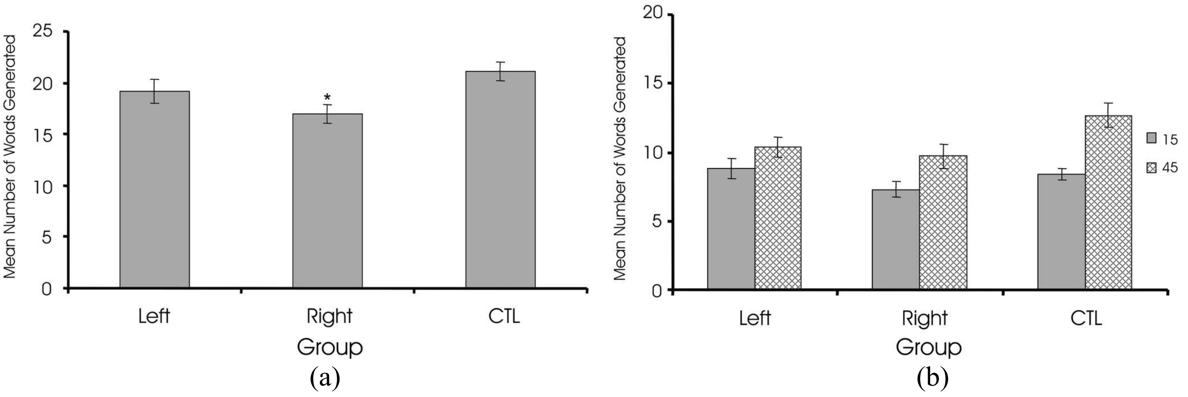

Fig. 4. (a) Total word generation in the semantic fluency task. (b) Word generation summed over three trials in the semantic fluency task, separated into time intervals defined by the first 15 seconds and the last 45 seconds.

of left prefrontal cortex and right cerebellum, perhaps primarily infero-lateral (e.g., crus I/II).

This result is in agreement with the weight of prior reports. Early case reports were mixed with results ranging from normal ([6] – posterior right lesion, 2 months post-onset) to mildly impaired ([25] – posterior right lesion, 5 weeks post-onset) to severely impaired ([14] – central right infarct, 2 weeks post onset). Of the group studies, several report reduced fluency, sometimes with a right laterality effect [15,18] but others less convincingly [22]. Richter and colleagues [18] found the greatest impairment in patients with lesions focused in the right posterior cerebellum (Crus II) and proposed that the right cerebellar lesion impaired "attention and inhibition" for a language-based task by disrupting functional connections to left prefrontal cortex. There have been other investigations that did not confirm an effect on fluency [7,11]. The reasons are not obvious – studied acutely, similar test demands, and in one [11] a preponderance of right postero-lateral lesions (PICA territory infarcts).

Leggio and colleagues [13] reported 25 patients, 19 with chronic focal lesions of various etiologies plus six degenerative disorders using a test and analysis strategy similar to ours. Both right and left focal groups were impaired on letter-based fluency but not on semantic fluency. The patients with degenerative disease were not impaired on total words on either task. No errors (perseverations or intrusions) were produced by any group. Patient groups showed reduced phonemic clustering on the letter-fluency task. The authors interpreted this deficit as an impairment in strategy application, emerging on phonemic tasks but not semantic ones because of the "novelty" of phonemic clustering.

Although there are discrepancies in the studies to date, a lesion of the right cerebellum is likely to impair verbal fluency, particularly letter fluency. The precise cognitive mechanism is not known but appears to be related to a domain specific executive/attentional deficit (see [23,24]). Whether that is best viewed as deficient task setting, deficient sustained attention or deficient strategy remains unclear. How those hypothetical mechanisms might be related themselves is also uncertain. That not all patients with right cerebellar lesions have this deficit, in fact in some studies even the group statistics do not reveal an effect, suggests that there may be a specific lesion site that the available lesion studies have not been able to disembed. The deep connectivity of the cerebellum is dauntingly tangled and complex (see [21], Fig. 4 for compelling visual support). At present it is only possible to observe that overall the lesion studies point to infero-lateral cerebellum – crusII – and the convergent neuroimaging studies point to the same area – lobule VI and crus I/crus II. Large studies that control for acuity, etiology and precise lesion site will clarify this issue.
